# Systematic identification of bacterial neuraminidase inhibitors from *Psoralea corylifolia* using ultrafiltration-UPLC-Q-Orbitrap-MS and molecular dynamics

**DOI:** 10.1080/14756366.2026.2676094

**Published:** 2026-05-25

**Authors:** Yu Jin Kim, Seol Jang, Youn-Hwan Hwang

**Affiliations:** a KM Convergence Research Division, Korea Institute of Oriental Medicine, Daejeon, Republic of Korea; b Research Infrastructure Team, Korea Institute of Oriental Medicine, Daejeon, Republic of Korea; c Korean Convergence Medical Science Major, KIOM Campus, University of Science & Technology (UST), Daejeon, Republic of Korea

**Keywords:** Bacterial neuraminidase, *Psoralea corylifolia*, UPLC–Q–Orbitrap–MS, enzyme kinetics, molecular dynamics

## Abstract

Bacterial neuraminidase (BNA) is a potential target for discovering anti-infective agents. Here, the BNA inhibitory activity of the ethanolic extract of *Psoralea corylifolia* (EEPC) was investigated using integrated experimental and computational approaches. Affinity ultrafiltration–UPLC–Q–Orbitrap–MS prioritised 11 representative constituents as putative BNA-interacting compounds. Five compounds (**7–11**) inhibited BNA with IC_50_ values of 4.67–34.09 μM. Bakuchiol (**11**) exhibited the strongest activity (IC_50_ = 4.67 ± 0.26 μM), and compounds **7**, **9**, and **10** were more active than the positive control, quercetin (IC_50_ = 13.63 ± 1.05 μM). Kinetic analysis indicated that bakuchiol (**11**) was competitive, whereas compounds **7–10** showed mixed-type inhibition. Docking and molecular dynamics analyses suggested that bakuchiol (**11**) and 3-hydroxybakuchiol (**10**) may form relatively stable BNA complexes. *In silico* absorption, distribution, metabolism, excretion, and toxicity analysis further supported bakuchiol (**11**) as a promising lead candidate among the tested compounds.

## GRAPHICAL_ABSTRACT

**Figure:**
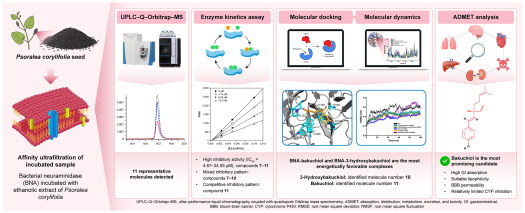

## Introduction

Bacterial neuraminidase (BNA) is a sialidase that cleaves terminal sialic acid residues from glycoproteins and glycolipids on host cell surfaces, thereby regulating host–pathogen interactions, biofilm formation, and bacterial pathogenicity^1^^,^^2^. Owing to its essential role in the colonisation and infection of target host cell surfaces, BNA has attracted increasing attention as a potential target for developing novel anti-infective agents^3^^,^^4^. While viral neuraminidase (NA) inhibitors, including oseltamivir^5^, zanamivir^6^, and peramivir^7^, are used in the treatment and prevention of influenza, research on BNA inhibitors remains relatively limited. In particular, existing viral NA inhibitors exhibit significantly reduced efficacy against BNA. For example, zanamivir is approximately a million-fold less effective against BNA than viral NA^8^. Furthermore, oseltamivir exerts limited effects on the growth and biofilm formation of bacteria such as *Streptococcus pneumoniae*^9^, highlighting the need for developing BNA-specific inhibitors. Recently, the BNA inhibitory effects of various phytochemicals, particularly phenolic compounds including flavonoids, chalcones, and xanthones, have been widely studied. Notably, compounds possessing prenyl substituents possess superior neuraminidase inhibitory activity^10–12^. However, most of these studies are limited to *in vitro* assays, enzyme kinetics, and molecular docking simulations. To the best of our knowledge, studies on the comprehensive dynamic interactions of enzyme–inhibitor complexes have not been reported.

The seed of *Psoralea corylifolia* (Fabaceae), also called “Bo-Gol-Zhee” in Korea, is a well-known traditional medicine that has been widely used since ancient times to treat various diseases, including vitiligo, alopecia areata, psoriasis, enuresis, urinary frequency, chills, and diarrhoea^13^^,^^14^. In addition, *P. corylifolia* possesses diverse pharmacological activities, including anti-inflammatory, antibacterial, antiviral, anticancer, antioxidant, cardioprotective, and immunomodulatory properties, and can effectively treat kidney, neurodegenerative, and musculoskeletal diseases^14^^,^^15^. More than 300 phytochemicals, including coumarins, flavonoids, meroterpenes, chalcones, and benzofurans, have been identified from *P. corylifolia*^16^. Their enzymatic activities, including the inhibitory effects of tyrosinase, acyl-CoA: cholesterol acyltransferase, protein tyrosine phosphatase 1B, α-glucosidase, and diacylglycerol acyltransferase, are well-established^17–19^. However, to our knowledge, the inhibitory effects and mechanisms of *P. corylifolia* constituents on BNA have not been investigated. Although the water extract of *P. corylifolia* exhibits inhibitory activity against viral NA^20^, the effects of its 70% ethanol extract and active constituents against BNA have not yet been investigated. As mentioned above, prenylated (C_5_) compounds exhibit BNA inhibitory activities^21–23^. Furthermore, given that various biological activities of *P. corylifolia* compounds containing diverse terpenoid moieties (including C_5_ prenyl and C_10_ geranyl/monoterpenoid groups) have been reported^24–26^, these constituents may possess inhibitory activity against BNA.

Affinity ultrafiltration combined with high-resolution liquid chromatography-mass spectrometry (LC-MS) is a powerful tool for screening potential bioactive compounds binding to target enzymes in complex natural products^27–29^. This high-throughput, rapid, sensitive, and convenient method is highly efficient for rapid screening of bioactive candidates in crude extracts and for subsequent evaluation of their interactions with target enzymes. Unlike conventional bioassay-guided screening, this approach enables rapid and simultaneous identification of active candidates in complex mixtures, significantly improving the efficiency of BNA inhibitor discovery. Subsequent studies on enzyme inhibitory activity and enzyme kinetics of the selected candidates have revealed their direct inhibitory potency and inhibition mechanism, respectively^30^. However, these approaches are insufficient to elucidate the underlying molecular mechanism causing inhibition; molecular docking analysis is required to predict interactions between the active site residues of enzymes and inhibitors^31^. To overcome the limitations of molecular docking, which statically represent the binding configuration, molecular dynamics (MD) simulations are performed, which provide a comprehensive understanding of the conformational stability, flexibility, and interactions of enzyme–ligand complexes under dynamic conditions over time^32^. In addition, *in silico* absorption, distribution, metabolism, excretion, and toxicity (ADMET) analysis is a useful approach for economically and rapidly predicting the pharmacokinetic and toxicological properties of bioactive candidates^33^. This integrated approach combines rapid ligand identification with dynamic interaction analysis and pharmacokinetic prediction, providing a more comprehensive evaluation of potential BNA inhibitors^34^.

In this study, we aimed to investigate the inhibitory potential of the ethanolic extract of *P. corylifolia* (EEPC) and its constituents against BNA from *Clostridium perfringens*, a gram-positive, spore-forming anaerobic bacterium^35^, using an integrated approach. First, we used affinity ultrafiltration combined with ultra-performance liquid chromatography coupled with quadrupole Orbitrap mass spectrometry (UPLC–Q–Orbitrap–MS) to screen for BNA-binding constituents in EEPC and selected 11 representative compounds. These compounds were evaluated for BNA inhibitory activity using an *in vitro* assay, and enzyme kinetic analysis was performed on five compounds (**7**–**11**) that exhibited high efficacy to determine the inhibition type and kinetic parameters. In addition, molecular docking was conducted to predict the binding mode of the BNA–ligand complex, and MD simulations were performed to elucidate the BNA–ligand interactions and molecular mechanism of inhibition through trajectory analyses. Furthermore, the pharmacokinetic and physicochemical suitability of the five potential candidates were assessed through *in silico* ADMET profiling. This integrated approach provides mechanistic and structural understanding of BNA inhibition by *P. corylifolia* constituents and is useful for studying their potential as candidates for BNA inhibitor development.

## Materials and methods

### Preparation of EEPC

The EEPC was prepared according as reported previously^36^. Briefly, 1 kg dried seeds of *P. corylifolia* (Gwangmyeongdang Pharm, Ulsan, Korea) were extracted by refluxing with 10 L of 70% (v/v) ethanol for 3 h. The extract was filtered, concentrated under reduced pressure, and lyophilised to yield 138.68 g (13.87%) of dried extract. The freeze-dried powder was stored at −20 °C until further use.

### Affinity ultrafiltration procedures

Affinity ultrafiltration was conducted as described previously, with slight modifications^22^, to identify BNA ligands by comparing the UPLC–Q–Orbitrap–MS profiles of the filtrates. Sodium acetate (Sigma-Aldrich, St. Louis, MO, USA) was dissolved in distilled water to prepare 50 mM sodium acetate buffer (pH 5.0), which was used throughout the experiments. Initially, 40 μL EEPC (50 mg/mL in dimethyl sulfoxide) was mixed with 710 μL of the buffer, vortexed for 10 min, and centrifuged at 14,000 rpm for 10 min. A 750 μL aliquot of the supernatant was pre-incubated at 37 °C for 1 h. BNA from *C. perfringens* (N2876; Sigma-Aldrich) was dissolved in the buffer to prepare a 0.5 U/mL solution. The pre-incubated EEPC solution was then mixed with 250 μL of BNA solution, yielding a final concentration of 2 mg/mL. For the BNA-free control, the BNA solution was replaced with an equal volume of buffer. The mixture was incubated at 37 °C for 0, 10, and 30 min on an incubator shaker (Lab Companion, Jeio Tech Co., Ltd., Daejeon, Korea) operating at 180 rpm. Following incubation, samples from each incubation time point, as well as the BNA-free control, were individually filtered through a 0.2 μm polytetrafluoroethylene hydrophobic membrane (Advantec, Tokyo, Japan) prior to UPLC–Q–Orbitrap–MS analysis, and the resulting filtrate containing unbound constituents was analysed. Potential active candidates were tentatively identified by comparing the chromatographic profiles of the filtrates with those of the corresponding control and monitoring decreases in peak intensities after incubation with BNA. Compounds in the filtrate showing reduced peak intensity were considered putative BNA-interacting candidates for further analysis, whereas those showing little or no change were considered less likely to interact under the assay conditions.

### UPLC–Q–Orbitrap–MS conditions

Reference standards of the 11 *P. corylifolia* compounds (purity ≥98%) were obtained from ChemFaces Biochemical Co., Ltd. (Wuhan, China). The chemical profiles of EEPC at different incubation times were analysed using a Dionex UltiMate 3000 UPLC system, which was interfaced with a Thermo Q–Exactive Orbitrap mass spectrometer (Thermo Fisher Scientific, Waltham, MA, USA) operating with an electrospray ionisation (ESI) source. Chromatographic separation was achieved using gradient elution with 0.1% (v/v) aqueous formic acid and acetonitrile on an Acquity BEH C_18_ column (100 × 2.1 mm, 1.7 µm, Waters, Milford, MA, USA) maintained at 40 °C. LC–MS grade solvents, including water, acetonitrile, and formic acid, were obtained from Thermo Fisher Scientific (Waltham, MA, USA). Mass spectrometric (MS) analysis was conducted with an ESI source in dual-polarity switching mode, and data were acquired in full MS–ddMS^2^ mode with normalised collision energy of 25 eV over a scan range of 100–1500 *m/z*. The negative ionisation data were used for ligand identification, as they provided superior sensitivity and clearer decreases in peak intensity the target constituents. Because the data were acquired in dual-polarity switching mode, no major constituent classes were excluded during acquisition; however, the negative mode was prioritised for interpretation because they provided more informative peak responses for the compounds of interest. Source parameters were optimised as follows: ion spray voltage was maintained at 3.8 kV, the capillary temperature was set at 320 °C, sheath and auxiliary gas pressures were 40 and 10 arbitrary units (au), respectively, and the S-lens RF level was 60. Resolution settings were 70,000 for full MS scans and 17,500 for ddMS^2^ scans. All data were acquired using Xcalibur v.3.0 and processed with FreeStyle v.1.8 (Thermo Fisher Scientific, Bremen, Germany).

### BNA inhibitory activity assay

BNA inhibitory activity was evaluated according to previously reported methods with slight modifications^37^. Sodium acetate buffer (50 mM, pH 5.0) was used as the reaction buffer, and 4-methylumbelliferyl-*N*-acetyl-α-d-neuraminic acid sodium salt hydrate (Sigma-Aldrich) was used as a fluorogenic substrate. The assay was performed in a total volume of 200 μL in a black 96-well microplate (SPL Life Science, Pocheon, Korea). The reaction mixture was prepared by mixing 160 μL of buffer with 20 μL of substrate (0.5 mM in buffer). Further, 10 μL of the inhibitor was added, followed by reaction initiation with 10 μL of BNA (0.05 units/mL in buffer). Fluorescence intensity was measured at excitation (365 nm) and emission (450 nm) wavelengths using a SpectraMax i3 microplate reader (Molecular Devices, San Jose, CA, USA) set at 37 °C. Half-maximal inhibitory concentration (IC_50_) values were determined via nonlinear regression analysis using SigmaPlot 15.0 (Systat Software Inc., San Jose, CA, USA).

### Enzyme kinetics assay

The enzyme kinetic parameters were determined by measuring the initial velocity at various substrate (0.25, 0.5, and 1.0 mM) and inhibitor concentrations. Five compounds were evaluated as inhibitors at concentrations of 0, 1.56, 3.12, and 6.25 μM for corylifol A (**7**) and bakuchiol (**11**); 0, 25, 50, and 100 μM for isoneobavaisoflavone (**8**); and 0, 3.12, 6.25, and 12.5 μM for 4′-*O*-methylbroussochalcone B (**9**) and 3-hydroxybakuchiol (**10**). The derived initial velocity results were used to identify the inhibition mechanism and kinetic constants by generating a Lineweaver–Burk plot (1/V vs. 1/[S]), where V and [S] represent the initial reaction rate and substrate concentration, respectively. This plot was used to identify the inhibition pattern and derive the kinetic constants. Secondary plots of the slope and y-intercept values versus inhibitor concentration were generated to calculate the inhibition constants, *K_i_* and *K_i′_*, corresponding to the inhibitor binding to the free enzyme and enzyme–substrate complex, respectively. All data were fitted using linear regression analysis with SigmaPlot 15.0 (Systat Software Inc., San Jose, CA, USA).

### Molecular docking analysis

Molecular docking simulations were performed to identify the interactions between key residues and ligands within the BNA catalytic site. The crystal structure of BNA (PDB ID: 2VK6) was obtained from the RCSB Protein Data Bank (https://www.rcsb.org)^38^. Prior to docking, the protein was preprocessed to remove water molecules and co-crystallized ligands using Discovery Studio 2025 (BIOVIA, Dassault Systèmes, San Diego, USA), and the ligand structure was energy minimised using the Universal Force Field in PyRx v.0.8^39^. The approximate coordinates of the binding pocket were determined using UCSF Chimaera 1.19^40^ based on the spatial positions of the co-crystallized ligands, which served as a reference for defining docking grid parameters in Discovery Studio. Docking simulations were performed using the AutoDock Vina algorithm in PyRx, with a grid box size of x = 30.28, y = 27.62, and z = 30.30 Å, centred at x = 2.57, y = 34.90, and z = 19.62 Å, including all key residues around the binding site, with an exhaustiveness value of 8 and a grid spacing of 1.0 Å. This grid box was defined to cover the catalytic pocket and adjacent surrounding residues, and the docking analysis was therefore followed a targeted docking approach rather than a blind search over the entire protein surface. The docking protocol was validated by redocking the native ligand (DAN) into the active site using the same parameters, and the root mean square deviation (RMSD) between the predicted pose and the native crystal structure was calculated using UCSF Chimaera 1.19. The interactions between BNA and ligands were visualised with the Discovery Studio 2025, which was used to generate 2D ligand–residue interaction diagrams and 3D representations of the BNA–ligand complexes.

### MD simulation

MD simulations were performed to validate the docking predictions and investigate the dynamic stability and conformational behaviour of the BNA–ligand complexes. All simulations were conducted for 100 ns using GROMACS 2025.1^41^. The topology and force field parameters for the ligands were generated using SwissParam in a manner compatible with the CHARMM36m force field used for BNA, and the systems were solvated with the SPC water model and neutralised with appropriate counterions. Energy minimisation was performed using the steepest descent algorithm until the maximum force converged to less than 1000 kJ/mol/nm. Equilibration was performed in the following two 100 ps sequential phases: NVT (V-rescale thermostat) and NPT (Berendsen barostat) maintained at 300 K and 1 bar. All bond lengths were constrained using the LINCS algorithm, and long-range electrostatic interactions were calculated using the Particle Mesh Ewald method with a cut-off distance of 1.2 nm. The integration time interval was 2 fs, and trajectory frames were stored every 1 ps. We assessed the structural stability and dynamics of the complex by performing trajectory analysis of conformational compactness, solvent accessibility, and local residue flexibility. To determine the persistence and energetic stability of the interaction between BNA and the ligand, hydrogen bond occupancy and interaction energy profiles were monitored for 100 ns. MD simulation data were used to identify major structural behaviours, and the conformational states were visualised using Discovery Studio 2025 and PyMol.

### Binding free energy calculation

Binding free energies (ΔG_total_) of the BNA–ligand complexes were estimated using the molecular mechanics/Poisson–Boltzmann surface area (MM/PBSA) method implemented in gmx_MMPBSA 1.6.4^42^. Representative snapshots were extracted every 50 ps from total 100 ns MD trajectories. The total binding free energy was calculated as follows: ΔG_total_ = ΔE_vdw_ + ΔE_ele_ + ΔG_solv_, where ΔE_vdw_ and ΔE_ele_ represent the van der Waals and electrostatic energy contributions in the gas phase, respectively, and ΔG_solv_ denotes the solvation free energy composed of polar and nonpolar components. The polar term was obtained using the Poisson–Boltzmann model, and the nonpolar term was derived from the solvent-accessible surface area (SASA) model. All computed energy terms were averaged to evaluate the relative contributions of nonpolar and polar interactions to the overall stabilisation of the complexes. The averaged free energy values were then used to compare the relative binding affinities among the ligand–enzyme complexes under identical simulation conditions.

### ADMET analysis

*In silico* ADMET analysis was performed to evaluate the pharmacokinetic and toxicity-related properties of the selected five potential candidates using the SwissADME web platform (http://www.swissadme.ch/)^43^. It predicted physicochemical, pharmacokinetic, and drug-like properties, including molecular weight, hydrogen bond (donors/acceptors), topological polar surface area (TPSA), lipophilicity (log *P*), gastrointestinal (GI) absorption, blood–brain barrier (BBB) permeability, water solubility, bioavailability score, cytochrome P450 (CYP) enzyme inhibition profiles, P-glycoprotein substrate status, and potential toxicity. Drug-likeness qualitatively assesses the potential of a molecule to be administered orally by evaluating its bioavailability according to Lipinski’s rule-of-five^44^ and Veber’s criteria^45^.

## Results and discussion

### UPLC–Q–Orbitrap–MS analysis

**Figure 1.**
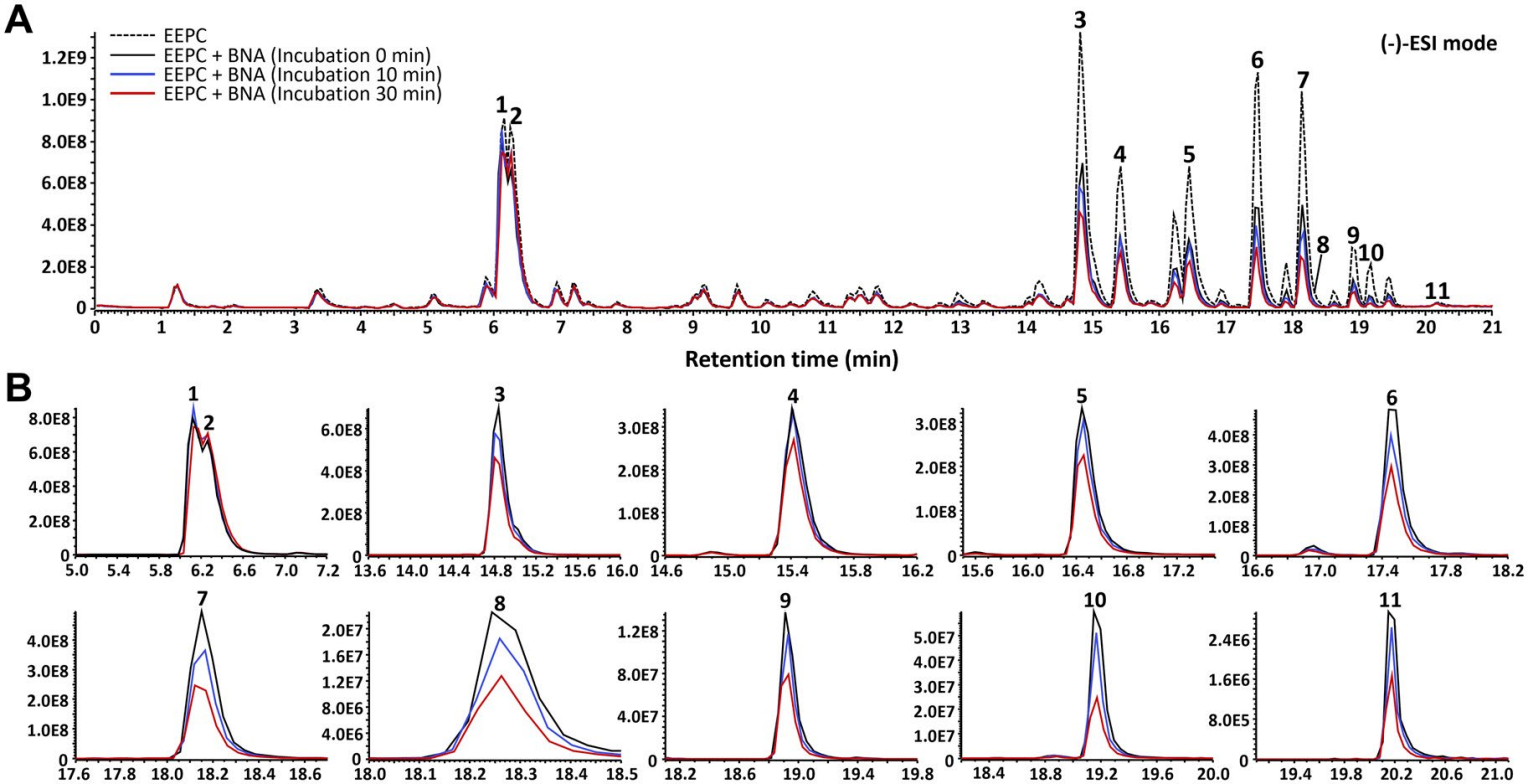
Affinity ultrafiltration–UPLC–Q–Orbitrap–MS analysis of ethanolic extract of *Psoralea corylifolia* (EEPC). (A) Base peak chromatograms of the EEPC filtrate without bacterial neuraminidase (BNA) compared with those of filtrates after 0, 10, and 30 min of incubation with BNA in negative ion mode. (B) Extracted ion chromatograms of constituents **1**–**11**, showing the time-dependent decrease in peak intensity during incubation with BNA.

EEPC contains various phytochemicals, including coumarins, flavonoids, chalcones, and meroterpenes, which exhibit diverse activities^16^. Affinity ultrafiltration–UPLC–Q–Orbitrap–MS was used to rapidly screen potential active compounds binding to BNA in complex EEPC. Samples incubated with BNA and EEPC for 0, 10, and 30 min, alongside a BNA-free control, were subjected to affinity ultrafiltration and then analysed using UPLC–Q–Orbitrap–MS to monitor changes in the peak intensity of each analyte (Figure 1A). The peak intensities in the BNA-free group were higher than those in all BNA-treated groups, suggesting that reduction in the intensity was associated with the presence of BNA rather than membrane filtration alone. Accordingly, 11 representative compounds were selected for further characterisation in the negative ion mode based on their peak intensities and differential peak intensity changes during incubation with BNA. In the extracted ion chromatograms, the analyte peak intensity gradually decreased with increasing incubation time (Figure 1B). Detailed MS characteristics, including the retention time, precursor ion, and fragment ion of the analytes, are summarised in Table 1. Compared with those in the initial sample (0 min), several compounds exhibited a decrease in peak intensity after 10 min of incubation, with most analytes showing a significant decrease by 30 min. Among the selected compounds, nine (**3**–**11**) were considered potential ligands because they showed distinct, time-dependent decreases in peak intensity after incubation with BNA. In contrast, two major compounds (**1** and **2**), which maintained stable and high peak intensities throughout the incubation period, were included as representative non-decreasing peaks for comparison. These findings suggest that the time-dependent decreases observed for compounds **3**–**11** were less likely to be explained by non-specific surface adsorption or generalised instability during incubation and were more closely associated with BNA-related interactions under the assay conditions. These results should be interpreted as indirect evidence of putative BNA-interacting constituents identified using the present screening workflow. Thus, this approach, combining affinity-based filtration and high-resolution MS in complex mixtures may facilitate the investigation of enzyme–ligand interactions and identification of potential active candidates.

**Table 1.** Identification of phytochemicals in EEPC by UPLC-Q-Orbitrap-MS.

No.	RT (min)	Adduct	Precursor ion (*m/z*)		Error (ppm)	Formula	MS/MS fragments (*m*/*z*)	Identification
			Calculated	Expected				
1	6.12	M-H	365.0876	365.0878	−0.5315	C_17_H_18_O_9_	203.0350, 159.0451	Psoralenoside
2	6.25	M-H	365.0877	365.0878	2.7364	C_17_H_18_O_9_	203.0350, 159.0450	Isopsoralenoside
3	14.84	M-H	321.1132	321.1132	−0.0305	C_20_H_18_O_4_	321.1133, 277.0509, 265.0504	Neobavaisoflavone
4	15.41	M-H	323.1290	323.1289	0.3646	C_20_H_20_O_4_	323.1288, 221.0819, 203.0714, 119.0501	Bavachin
5	16.45	M-H	335.0926	335.0925	0.2696	C_20_H_16_O_5_	335.0926, 312.0273, 280.0376	Psoralidin
6	17.44	M-H	323.1289	323.1289	−0.0132	C_20_H_20_O_4_	323.1289, 203.0714, 119.0501	Isobavachalcone
7	18.15	M-H	389.1760	389.1758	0.3452	C_25_H_26_O_4_	389.1756, 320.1057, 303.1015, 277.0498, 265.0503	Corylifol A
8	18.24	M-H	321.1133	321.1132	0.0646	C_20_H_18_O_4_	321.1132, 201.0558, 119.0501	Isoneobavaisoflavone
9	18.91	M-H	337.1448	337.1445	0.7278	C_21_H_22_O_4_	337.1445, 293.1549, 119.0501	4′-O-Methylbroussochalcone B
10	19.15	M-H	271.1705	271.1704	0.5595	C_18_H_24_O_2_	271.1703, 256.1469, 188.0842	3-Hydroxybakuchiol
11	20.15	M-H	255.1757	255.1754	0.8589	C_18_H_24_O	255.1754, 213.0547, 172.0894	Bakuchiol

### BNA inhibitory activity

**Figure 2.**
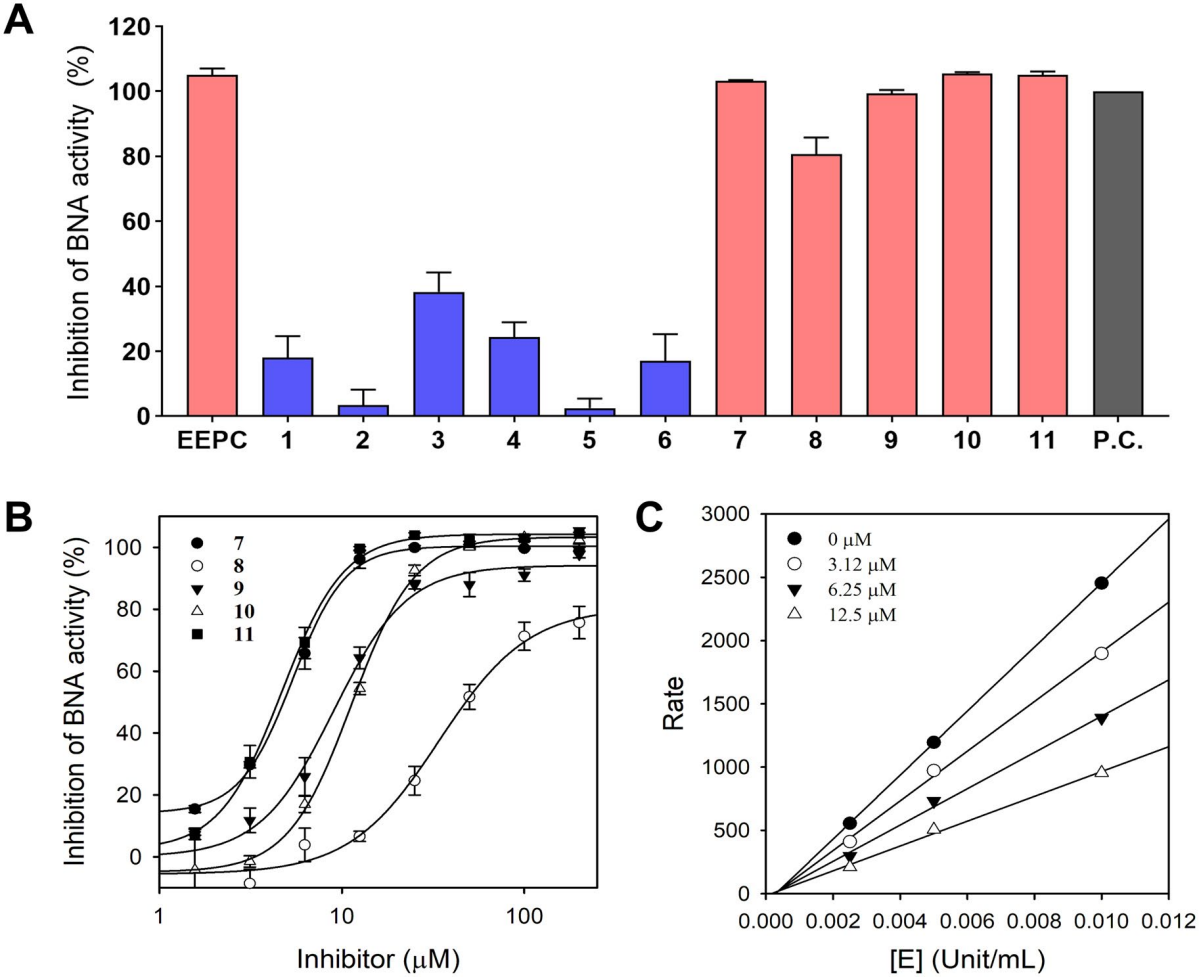
Inhibitory effects of ethanolic extract of *Psoralea corylifolia* (EEPC) constituents against bacterial neuraminidase (BNA). (A) BNA inhibitory activities of EEPC and 11 compounds (**1**–**11**) at 100 μM. Quercetin was used as a positive control and its inhibitory activity was set to 100%. (B) Dose-response curves of compounds **7**–**11** fitted via nonlinear regression for IC_50_ determination. (C) Enzyme concentration-dependence of BNA catalytic activity at various concentrations of compound **9**.

**Figure 3.**
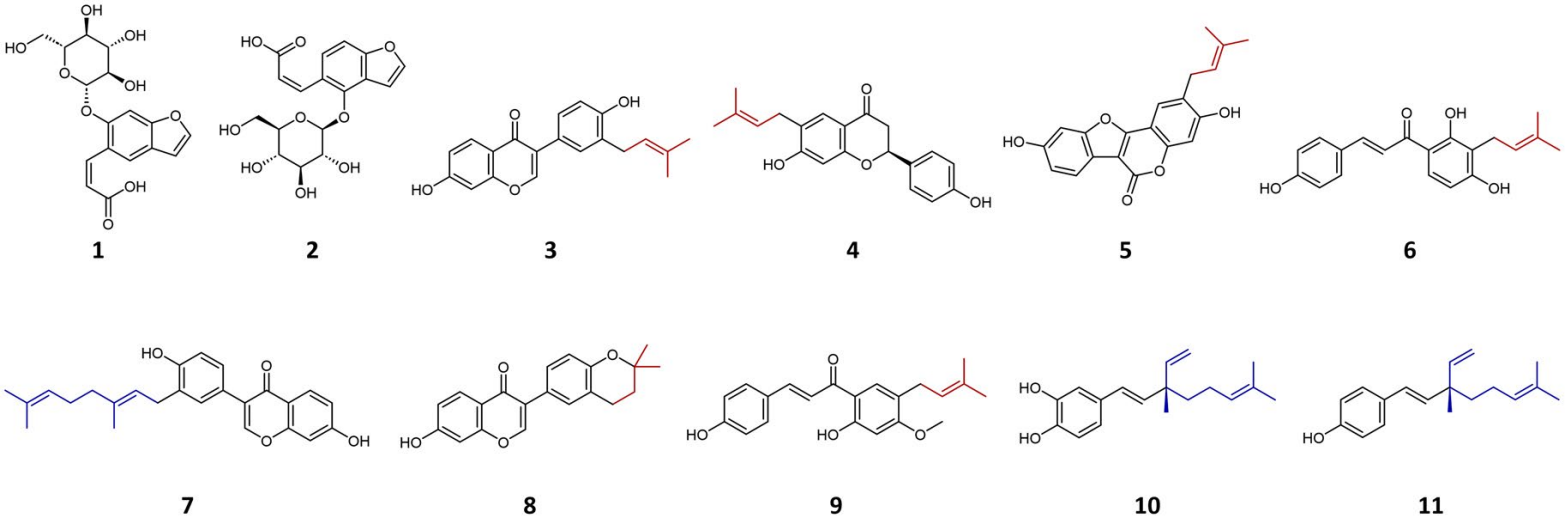
Chemical structures of compounds **1**–**11**. The C_5_ prenyl (red) and C_10_ geranyl or monoterpenoid (blue) substituents, including the cyclized C_5_ moiety in compound **8**, are highlighted.

The inhibitory effects of EEPC and the 11 selected representative constituents on BNA were evaluated at 100 μM, with quercetin used as a positive control^46^. All values were normalised to the positive control set to 100% inhibition. EEPC showed an inhibitory level similar to that of the positive control (Figure 2A). Compounds **1**–**6** exhibited weak effects with less than 50% inhibition. In contrast, corylifol A (**7**), 4′-*O*-methylbroussochalcone B (**9**), 3-hydroxybakuchiol (**10**), and bakuchiol (**11**) exhibited potent inhibitory activities comparable to the positive control; isoneobavaisoflavone (**8**) also showed an inhibition level exceeding 80%. Consequently, the dose-dependent inhibition (%) of compounds **7**–**11** was evaluated via nonlinear regression analysis (Figure 2B) and IC_50_ values (Table 2). Based on the IC_50_ values, corylifol A (**7**) (IC_50_ = 5.36 ± 0.19 μM), 4′-*O*-methylbroussochalcone B (**9**) (IC_50_ = 8.92 ± 0.68 μM), 3-hydroxybakuchiol (**10**) (IC_50_ = 11.27 ± 0.33 μM), and bakuchiol (**11**) (IC_50_ = 4.67 ± 0.26 μM) exhibited more potent inhibitory activity than the positive control, quercetin (IC_50_ = 13.63 ± 1.05 μM). However, isoneobavaisoflavone (**8**) (IC_50_ = 34.09 ± 4.66 μM) showed relatively moderate activity. The structural features of these compounds are presented in Figure 3. Although prenylated (C_5_) moieties are widely recognised for their BNA inhibitory effects^21–23^, our results suggest that inhibitory efficacy may be influenced by the configuration and chain length of these terpenoid substituents. While compounds **3**–**6** possess a typical 3,3-dimethylallyl group (C_5_) on their backbones but showed weak inhibitory activity, isoneobavaisoflavone (**8**), containing a cyclized C_5_ prenyl group (2,2-dimethylchroman moiety), showed moderate inhibitory activity. Furthermore, most of the more active inhibitors (**7, 10,** and **11**) were characterised by longer C_10_ geranyl or monoterpenoid chains, whereas 4′-*O*-methylbroussochalcone B (**9**) achieved high potency through its specific C_5_ prenylation on the chalcone scaffold. In summary, our findings suggest that BNA inhibitory efficacy is not solely determined by the presence of a prenyl group, but may also be influenced by the structural type, chain length (C_5_ or C_10_), and cyclisation of the terpenoid moiety.

**Table 2.** IC_50_, *K_i_*, and *K_i′_* values of EEPC compounds against BNA

No.	IC_50_^a^ (μM)	Inhibition type	(*K_i_* ^b^, μM)	(*K_i′_*^c^, μM)
1	> 100	Not tested	–	–
2	> 100	Not tested	–	–
3	> 100	Not tested	–	–
4	> 100	Not tested	–	–
5	> 100	Not tested	–	–
6	> 100	Not tested	–	–
7	5.36 ± 0.19	Mixed type	1.00 ± 0.29	7.16 ± 0.44
8	34.09 ± 4.66	Mixed type	58.63 ± 5.89	149.68 ± 7.54
9	8.92 ± 0.68	Mixed type	5.76 ± 0.65	43.72 ± 5.04
10	11.27 ± 0.33	Mixed type	5.44 ± 0.63	38.42 ± 3.17
11	4.67 ± 0.26	Competitive	1.64 ± 0.52	–
Quercetin^d^	13.63 ± 1.05	Not tested	–	–

All data are presented as mean ± standard deviation (SD) from triplicate experiments.

^a^IC_50_ values indicate the inhibitor concentration required to reduce bacterial neuraminidase activity by 50%.

^b^*K_i_* represents the inhibition constant for inhibitor binding to the free enzyme.

^c^*K_i′_* represents the inhibition constant for inhibitor binding to the enzyme-substrate complex.

^d^Positive control.

Notably, bakuchiol (**11**) exhibited potent BNA inhibitory activity (IC_50_ = 4.67 ± 0.26 μM), which was within the range reported for anti-influenza activities of (+)-(*S*)-bakuchiol (IC_50_ = 0.2–13.9 μM) in cell-based assays^47^^,^^48^. Although these reported values were not derived from direct NA inhibition assays, they provide additional context for the biological activity of bakuchiol (**11**).

### Determination of inhibition mechanism for the five active compounds

The dependency of BNA activity on enzyme concentration was investigated for five active compounds, and the results for 4′-*O*-methylbroussochalcone B (**9**) are representatively shown in Figure 2C. The reaction rate increased linearly with enzyme concentration and reached a steady state. The slope of the linear curve decreased with increasing concentration of 4′-*O*-methylbroussochalcone B (**9**). The remaining compounds also inhibited BNA activity in a concentration-dependent manner, suggesting that all five compounds acted as reversible inhibitors.

**Figure 4.**
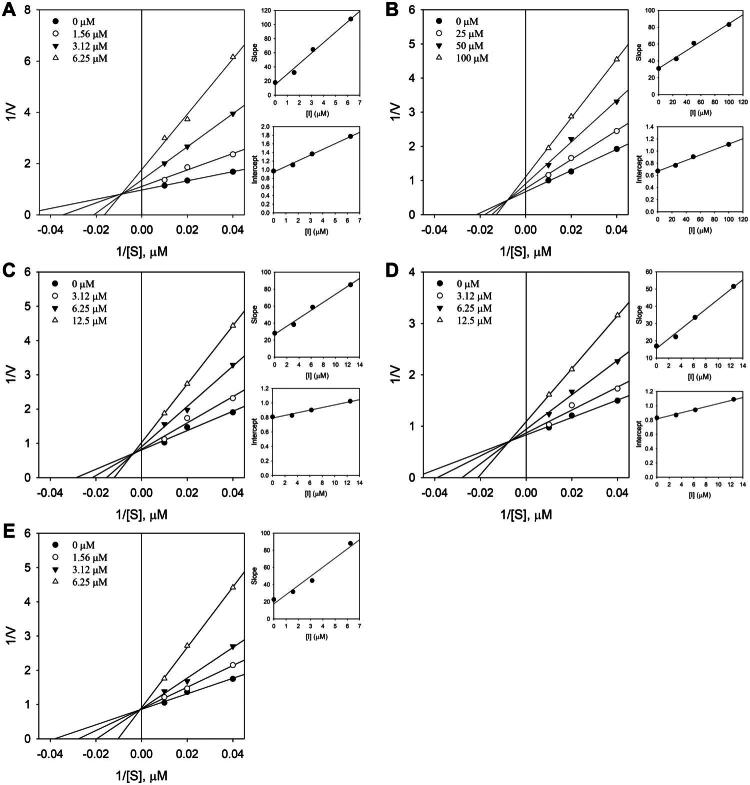
Enzyme kinetic analysis of bacterial neuraminidase (BNA) inhibition by compounds **7**–**11**. (A)–(E) Lineweaver–Burk plots for compounds **7**–**11**. Secondary plots of slopes and Y-intercepts against inhibitor concentration were used to determine the inhibition constants (*K_i_* and *K_i′_*).

Enzyme kinetics was analysed to investigate the inhibitory mechanisms of corylifol A (**7**), isoneobavaisoflavone (**8**), 4′-*O*-methylbroussochalcone B (**9**), 3-hydroxybakuchiol (**10**), and bakuchiol (**11**) against BNA, and the initial reaction rates were measured at various concentrations of each compound using substrate concentrations of 0.25, 0.5, and 1.0 mM. In the generated Lineweaver–Burk double-reciprocal plots (1/V vs. 1/[S]), the inhibition pattern was determined from the intersection of the linear regression lines. Consequently, corylifol A (**7**), isoneobavaisoflavone (**8**), 4′-*O*-methylbroussochalcone B (**9**), and 3-hydroxybakuchiol (**10**) exhibited mixed-type inhibition, characterised by lines intersecting to the left of the y-axis and above the x-axis (Figure 4A–D), suggesting that these compounds interact with both the free enzyme and the enzyme–substrate complex. In contrast, bakuchiol (**11**) showed competitive inhibition, wherein the lines intersected at the y-axis, indicating that the inhibitor directly competes with the substrate for binding to the active site (Figure 4E). The *K_i_* and *K_i′_* were determined from the slope and y-intercept derived from a Lineweaver–Burk plot versus inhibitor concentration in a secondary plot^49^^,^^50^. The calculated inhibition constants for the five compounds are summarised in Table 2, and the corresponding secondary plots are presented in Figure 4A–E. For corylifol A (**7**), isoneobavaisoflavone (**8**), 4′-*O*-methylbroussochalcone B (**9**), and 3-hydroxybakuchiol (**10**), which showed mixed-type inhibition, the *K_i_* and *K_i′_* values were determined from the slope and intercept plots, respectively, representing binding to the free enzyme and enzyme–substrate complex, respectively. Notably, all four compounds exhibited *K_i′_* values higher than their corresponding *K_i_* values, suggesting relatively weaker interactions with the enzyme–substrate complex. In contrast, bakuchiol (**11**), which showed competitive inhibition, demonstrated selective binding to the free enzyme, as reflected by the *K_i_* value derived solely from the slope plot. Collectively, the kinetic analysis suggests that structural differences among the five compounds lead to different binding modes towards BNA, resulting in two different inhibitory mechanisms. All kinetic experiments were performed in triplicate, and kinetic parameters are expressed as the mean ± standard deviation.

### Molecular docking analysis of BNA–ligand complexes

**Figure 5.**
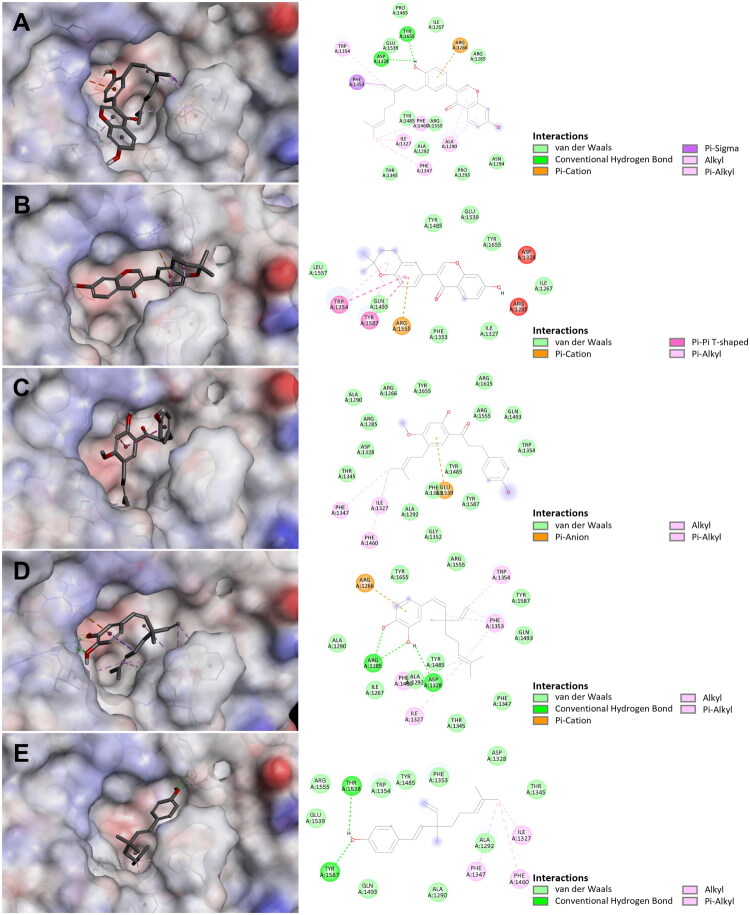
Predicted binding modes of compounds **7**–**11** within the catalytic pocket of bacterial neuraminidase (BNA). (A)–(E) Docking poses of compounds **7**–**11**, with key residues forming hydrogen bonds, π-π interactions, and hydrophobic contacts indicated.

The docking protocol was validated through redocking of the co-crystallized ligand (DAN) into the active site of BNA (PDB ID: 2VK6), which yielded an RMSD of 0.36 Å, supporting the reliability of the docking settings used in this study. Molecular docking simulations were performed to visualise the binding modes and quantitatively assess the five active compounds (**7**–**11**). The predicted binding affinities were determined as follows: corylifol A (**7**) (−7.9 kcal/mol), isoneobavaisoflavone (**8**) (−7.8 kcal/mol), 4′-*O*-methylbroussochalcone B (**9**) (−7.4 kcal/mol), 3-hydroxybakuchiol (**10**) (−6.8 kcal/mol), and bakuchiol (**11**) (−7.0 kcal/mol). All five compounds were predicted to occupy poses within the catalytic pocket of 2VK6, the DAN binding site, which is surrounded by the key catalytic residues ARG1266/1555, TYR1485/1655, ASP1328, and GLU1539 involved in enzyme catalysis^22^. All compounds were predicted to interact with surrounding residues through hydrogen bonds, hydrophobic contacts, and π-π stacking interactions, which may contribute to ligand accommodation within the catalytic region (Figure 5A–E).

The terpenoid moieties (C_5_ prenyl and C_10_ geranyl/monoterpenoid groups) of corylifol A (**7**), 4′-*O*-methylbroussochalcone B (**9**), 3-hydroxybakuchiol (**10**), and bakuchiol (**11**) showed similar interaction patterns, serving as hydrophobic groups positioned deeply within the catalytic pocket. These side chains interacted with key residues, including PHE1347, PHE1460, and ILE1327. Notably, in 3-hydroxybakuchiol (**10**), the interaction occurred with PHE1353 instead of PHE1347. Corylifol A (**7**) formed hydrogen bonds with ASP1328 and TYR1655, alongside hydrophobic contacts with ARG1266, PHE1353, ALA1290, and TRP1354. 4′-*O*-Methylbroussochalcone B (**9**) showed a π-anion interaction with GLU1539 and van der Waals interactions with residues such as PHE1353, ARG1266, ASP1328, and TYR1485, which may contribute to binding within the catalytic region. 3-Hydroxybakuchiol (**10**) was predicted to interact within the pocket through hydrogen bonds with ARG1285 and ASP1328, a π-cation contact with ARG1266, a π-alkyl interaction with TRP1354, and van der Waals interactions with nearby residues. Bakuchiol (**11**) formed hydrogen bonds with THR1538 and TYR1587, complemented by surrounding van der Waals interactions. Consequently, the deeply inserted terpenoid chains of corylifol A (**7**), 4′-*O*-methylbroussochalcone B (**9**), 3-hydroxybakuchiol (**10**), and bakuchiol (**11**) may contribute to hydrophobic contacts within the catalytic region and may be associated with their relatively stronger inhibitory activities compared with isoneobavaisoflavone (**8**), which contains a cyclized C_5_ prenyl group.

Overall, the docking results showed broad agreement with the experimental inhibition trends in terms of the binding orientations and favourable affinities at the catalytic site. Compared with the other compounds, bakuchiol (**11**) interacted more deeply within the catalytic pocket, which may be consistent with the experimentally observed competitive inhibition mechanism. For the mixed-type inhibitors corylifol A (**7**), isoneobavaisoflavone (**8**), 4′-*O*-methylbroussochalcone B (**9**), and 3-hydroxybakuchiol (**10**), the kinetic analysis revealed that their *K_i_* values (binding to free enzyme) were consistently lower than their *K_i′_* values (binding to ES complex), suggesting a stronger interaction with the free enzyme than with the ES complex. In this context, the docking results provide a plausible structural model for the free-enzyme binding component within or near the catalytic pocket. However, because the present docking analysis was targeted to the catalytic region rather than it being a full blind-docking search across the entire protein surface, it did not exclude additional binding events outside the catalytic pocket or interactions with the ES complex that may contribute to the mixed-type inhibition pattern. Although docking scores did not perfectly correlate with IC_50_ values, the overall alignment with the kinetic data supports the mechanistic relevance of the predicted binding poses.

### MD simulations of BNA–ligand complexes

MD simulations were performed to further investigate the interactions between BNA and the ligands corylifol A (**7**), isoneobavaisoflavone (**8**), 4′-*O*-methylbroussochalcone B (**9**), 3-hydroxybakuchiol (**10**), and bakuchiol (**11**). Whereas molecular docking statically represents binding configurations, MD simulations capture the time-dependent features of enzyme–ligand interactions, allowing assessment of trajectory stability and binding thermodynamics. In this study, various computational metrics were integrated to obtain complementary insights into the inhibitory mechanisms. In particular, structural stability and compactness were assessed using RMSD, radius of gyration (Rg), and SASA, whereas local flexibility and polar contacts were assessed via root mean square fluctuations (RMSF) and hydrogen-bond persistence. Furthermore, free energy landscape (FEL) and MM/PBSA-based binding free energy were combined to correlate conformational dynamics with thermodynamic binding affinities, providing additional context for the observed inhibitory activities.

#### Structural stability and dynamics

**Figure 6.**
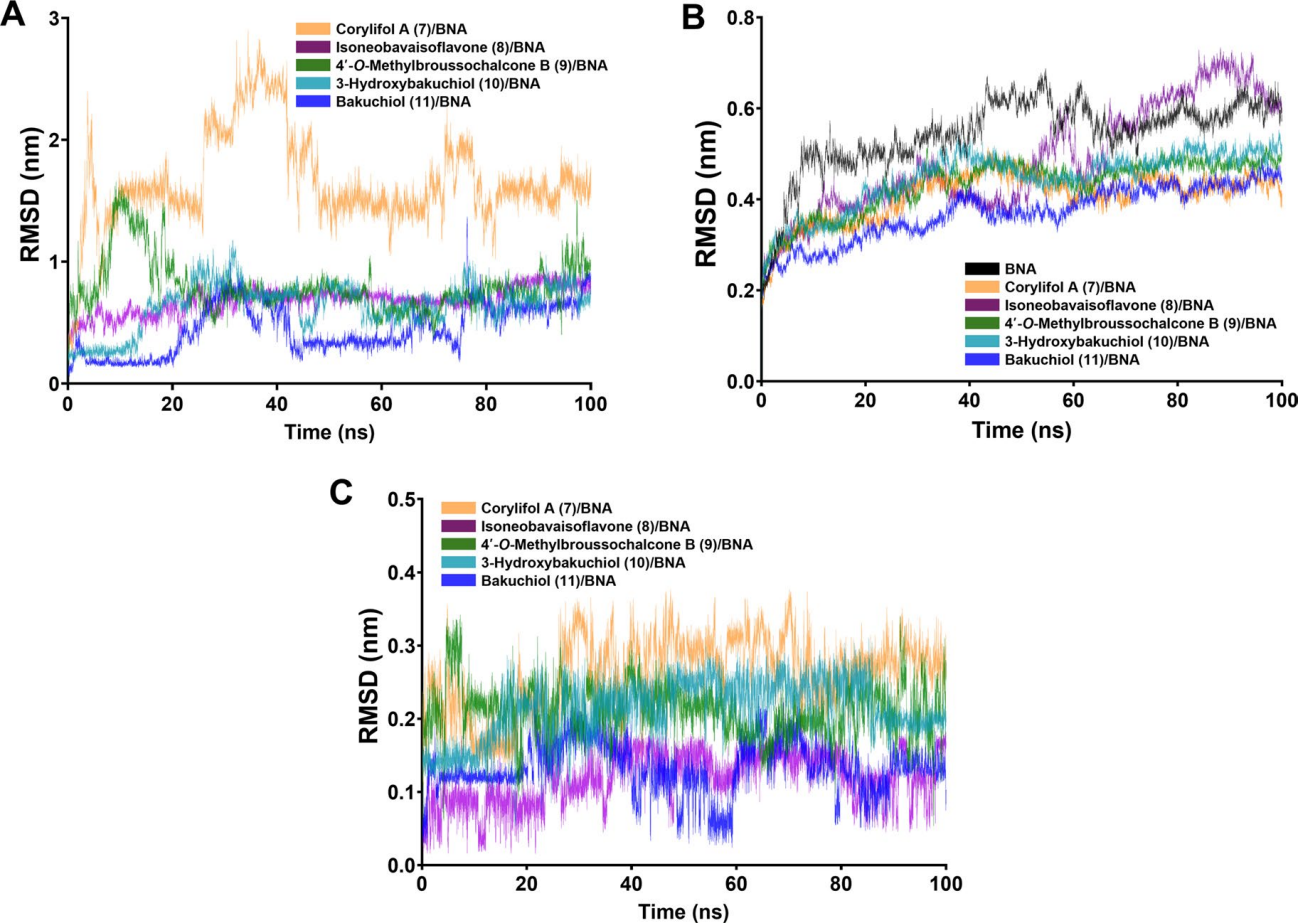
Root mean square deviation (RMSD) profiles of bacterial neuraminidase (BNA)–ligand complexes during 100 ns molecular dynamics (MD) simulations. (A) RMSD of BNA–ligand (**7**–**11**) complexes. (B) RMSD of BNA backbone of free BNA and BNA–ligand complexes. (C) Ligand RMSD of compounds **7**–**11**.

The dynamic stability of BNA–ligand complexes was evaluated via 100 ns MD simulations^32^. The ligand RMSD profiles (Figure 6A) revealed that most complexes reached a stable equilibrium after slight initial fluctuations during the first 30–40 ns. Isoneobavaisoflavone (**8**), 4′-*O*-methylbroussochalcone B (**9**), 3-hydroxybakuchiol (**10**), and bakuchiol (**11**) maintained low RMSD values (<1 nm), suggesting stable binding within the catalytic pocket. In contrast, corylifol A (**7**) exhibited relatively larger fluctuations, likely due to the structural flexibility of its bent flavonoid backbone rather than structural instability. The BNA backbone RMSD (Figure 6B) stabilised within the 0.3–0.5 nm range. Notably, the backbone fluctuations of all complexes were slightly lower than those of the free BNA, suggesting that ligand binding marginally enhances BNA stability. Among them, bakuchiol (**11**), the most potent inhibitor, exhibited the lowest backbone RMSD, suggesting a possible relationship between its high inhibitory potency and the structural stability of the complex. Corylifol A (**7**), isoneobavaisoflavone (**8**), 4′-*O*-methylbroussochalcone B (**9**), and 3-hydroxybakuchiol (**10**) generally showed stable trajectories, with only minor late-stage fluctuations observed for isoneobavaisoflavone (**8**). Positional stability was assessed to evaluate the inherent flexibility of each ligand within the binding site (Figure 6C). All ligands exhibited RMSD values <0.3 nm, with those maintaining approximately 0.1 nm showing high positional stability. These results suggest that all BNA–ligand complexes maintained relatively stable conformations throughout the simulation without inducing significant structural changes in the overall BNA.

Structural compactness and solvent accessibility of the BNA–ligand complexes were evaluated through Rg and SASA analyses^51^. As shown in Supplementary Fig. S2A, all BNA–ligand complexes maintained stable Rg values within a narrow range (2.2–2.3 nm), indicating that ligand binding did not induce significant large-scale expansion or contraction of the BNA structure. Notably, corylifol A (**7**) exhibited a slightly lower average Rg value than that of the free BNA, indicating an increase in structural compactness upon binding. Regarding the SASA profiles (Supplementary Fig. S2B), all complexes exhibited stable SASA values throughout the 100 ns simulation, with average values ranging from approximately 190 to 220 nm^2^. The corylifol A (**7**) complex consistently exhibited the lowest SASA values, suggesting reduced solvent exposure and a more compact structural arrangement of the catalytic pocket. Although the full 0–100 ns trajectories are shown to illustrate the overall structural evolution during the simulation, the interpretation of the Rg and SASA profiles was based primarily on the stabilised interval from 50 to 100 ns. The consistent trends in Rg and SASA across all complexes suggest that ligand binding minimises structural fluctuations and stabilises the BNA structure by reducing solvent exposure. Overall, the comprehensive analysis of RMSD, Rg, and SASA indicates relatively high dynamic stability of all the BNA–ligand complexes. These results suggest that the complexes consistently maintained structural stability and compactness throughout the 100 ns simulation under physiological conditions.

#### Residual flexibility and hydrogen-bond persistence

The local flexibility of the BNA structure was evaluated through RMSF analysis to quantify the positional fluctuations of individual residues^52^. All BNA–ligand complexes exhibited generally similar RMSF profiles, indicating that the overall fold of the BNA remained stable upon binding (Supplementary Fig. S3A). However, specific regions, approximately 1350–1400, 1550–1600, and 1620–1660, exhibited noticeable differences in flexibility among the complexes. The most potent inhibitors, bakuchiol (**11**) and corylifol A (**7**), exhibited lower average RMSF values in these fluctuating regions compared to the free BNA and other complexes, suggesting limited flexibility and greater structural stability of the residues surrounding the binding site throughout the simulations. In contrast, isoneobavaisoflavone (**8**) and 4′-*O*-methylbroussochalcone B (**9**) showed increased RMSF values in specific regions, such as around residues 1360–1400 for isoneobavaisoflavone (**8**) and around 1550–1600 for 4′-*O*-methylbroussochalcone B (**9**). These observations may reflect increased conformational flexibility within the pocket, suggesting that these ligands undergo greater structural adjustments to maintain stable binding.

The stability and persistence of the interactions between BNA and each ligand were evaluated through hydrogen-bond profiles. In general, a greater number and higher persistence of hydrogen bonds tend to reflect stronger bonding stability, potentially contributing to higher inhibitory potential^52^. All complexes exhibited dynamic hydrogen-bonding patterns, maintaining between zero and four bonds throughout the 100 ns simulation (Supplementary Fig. S3B). Among the ligands, bakuchiol (**11**) and 3-hydroxybakuchiol (**10**) showed the most persistent hydrogen bonds, generally maintaining more than two bonds. In particular, 3-hydroxybakuchiol (**10**) intermittently exhibited up to four hydrogen bonds, suggesting a robust interaction with BNA. In contrast, 4′-*O*-methylbroussochalcone B (**9**) formed many hydrogen bonds early in the simulation but later showed transiently maintained bonds with large fluctuations (1–3), indicating a partially stabilised binding profile. Furthermore, corylifol A (**7**) and isoneobavaisoflavone (**8**) exhibited fewer hydrogen bonds compared with the other complexes. Corylifol A (**7**) formed intermittent and transient hydrogen bonds throughout the trajectory, whereas isoneobavaisoflavone (**8**) showed a gradual adaptation within the binding pocket, establishing more persistent bonds after the initial 30 ns. These results suggest that while polar interactions are restricted for these ligands, the overall complex stability is likely driven by non-polar interactions, such as hydrophobic effects or π-π stacking, which compensate for the limited hydrogen bonding.

#### Interaction energy

The stability of the BNA–ligand complexes was further investigated by analysing non-bonded interaction energies, specifically electrostatic (Coulombic short-range, Coul-SR) and van der Waals (Lennard–Jones short-range, LJ-SR) contributions^53^. All BNA–ligand complexes maintained negative Coul-SR interaction energies throughout the 100 ns simulation, indicating favourable electrostatic interactions (Supplementary Fig. S4A). Bakuchiol (**11**) and 3-hydroxybakuchiol (**10**) exhibited the most significant negative Coul-SR energies. 4′-*O*-Methylbroussochalcone B (**9**) also showed favourable electrostatic interaction (approximately −150 kJ/mol), although the variability of the energy values was relatively large compared with that of the other complexes. In contrast, corylifol A (**7**) and isoneobavaisoflavone (**8**) showed relatively weaker and less stable electrostatic interactions, with approximate values of −50 kJ/mol.

Nonpolar stabilisation was assessed via LJ-SR interaction energy analysis (Supplementary Fig. S4B). All complexes consistently exhibited negative LJ-SR energies ranging from −50 to −150 kJ/mol, suggesting that van der Waals interactions significantly stabilised the complexes. Notably, the LJ-SR energy for isoneobavaisoflavone (**8**) was slightly lower than that of the other complexes towards the end of the simulation, indicating an enhanced van der Waals contribution. Overall, the LJ-SR interactions displayed more negative energy values than the Coul-SR interactions, suggesting that van der Waals forces may exert a greater influence than electrostatic interactions on the stability of all complexes.

#### Free energy landscape analysis

**Figure 7.**
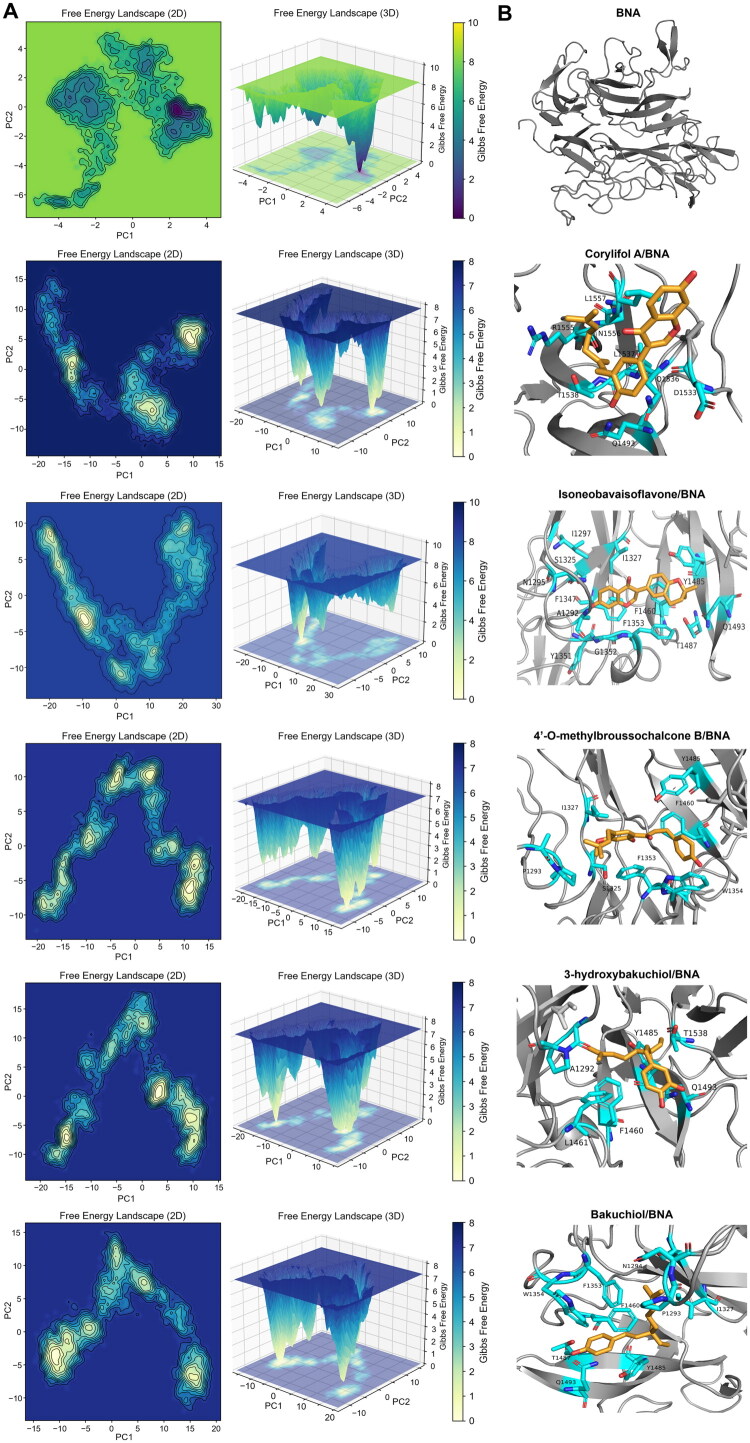
Free energy landscape (FEL) and representative structures of free bacterial neuraminidase (BNA) and BNA–ligand (**7**–**11**) complexes. (A) 2D and 3D FELs constructed from the principal components 1 and 2 (PC1 and PC2) of 100 ns MD trajectories for free BNA and BNA–ligand complexes. (B) Representative low-energy conformations extracted from the main FEL minima.

The conformational dynamics and stability of the free BNA and BNA–ligand complexes were evaluated through principal component analysis (PCA) to construct 2D and 3D FEL surfaces (Figure 7A). Deep valleys in the 3D FEL indicate low Gibbs free energies and stable conformations, whereas broad and shallow regions indicate higher free energy and increased structural flexibility^54^. Free BNA exhibited a relatively broad and shallow energy distribution, indicating a highly flexible conformational state and suggesting that the unbound enzyme can dynamically adapt to the binding of various ligands. Upon ligand binding, the energy landscapes generally became more compact, indicating that each ligand is anchored within the catalytic pocket through stable polar and hydrophobic interactions (Figure 7B).

The FEL profiles of the bakuchiol (**11**) and 3-hydroxybakuchiol (**10**) complexes exhibited the deepest and most distinct minima, suggesting highly stable conformational states with minimised energy fluctuations. The 4′-*O*-methylbroussochalcone B (**9**) complex also showed deep energy minima and several moderate energy regions distributed within a narrow conformational range, indicating a stable binding mode with limited flexibility. The corylifol A (**7**) complex exhibited three distinct local minima, suggesting a dynamically stable configuration that allows for structural fluctuations. Notably, the isoneobavaisoflavone (**8**) complex showed a relatively broad and shallow energy region; although it contained a single low-energy minimum, the overall landscape remained dispersed, reflecting more flexible interactions and increased dynamic fluctuations compared with those of the other ligands.

In summary, bakuchiol (**11**) and 3-hydroxybakuchiol (**10**) apparently formed the most energetically stable complexes with BNA. In contrast, corylifol A (**7**) and 4′-*O*-methylbroussochalcone B (**9**) exhibited moderate stability, reflecting localised conformational flexibility. The FEL of the isoneobavaisoflavone (**8**) complex showed a broad energy distribution, suggesting the most flexible interaction among the five ligands. Overall, FEL analysis revealed that ligand binding modulated the structural energy landscape of BNA, providing molecular insights into the diverse binding modes and structural stability of the complexes.

#### Binding free energy analysis

The binding free energies of the five ligand–BNA complexes were quantitatively evaluated using MM/PBSA calculations (Table 3). To ensure that only equilibrated conformations were used, the MM/PBSA binding free energy calculations were performed using the final 50 ns of each trajectory (50–100 ns), as defined from the RMSD stabilisation profiles. The total binding free energy (ΔG_total_) is the sum of the gas-phase interaction energy (ΔG_gas_), which includes van der Waals (ΔE_vdw_) and electrostatic (ΔE_ele_) interactions energies, and the solvation free energy (ΔG_solv_); this parameter is used to evaluate the thermodynamic stability of each complex^55^. MM/PBSA analysis revealed that isoneobavaisoflavone (**8**) exhibited the most favourable total binding free energy (ΔG_total_ = −20.22 ± 4.10 kcal/mol), followed closely by bakuchiol (**11**) (ΔG_total_ = −15.77 ± 4.44 kcal/mol) and 3-hydroxybakuchiol (**10**) (ΔG_total_ = −15.54 ± 3.24 kcal/mol). Van der Waals interactions played a dominant role in stabilising all complexes, particularly for isoneobavaisoflavone (**8**) (−31.86 kcal/mol). Although ΔG_solv_ values were positive for all complexes, acting as an energetic penalty, the strong van der Waals interactions compensated for the significant desolvation, especially for isoneobavaisoflavone (**8**) (ΔG_solv_ = 22.65 ± 3.46 kcal/mol).

**Table 3.** Binding free energy (MM/PBSA) analysis of BNA–ligand complexes (kcal/mol).

Complex	ΔE_vdw_	ΔE_ele_	ΔG_gas_	ΔG_solv_	ΔGtotal
Corylifol A/BNA	−23.29 ± 3.25	−1.11 ± 2.83	−24.39 ± 4.46	10.04 ± 3.78	−14.35 ± 2.60
Isoneobavaisoflavone/BNA	−31.86 ± 1.84	−11.00 ± 4.01	−42.86 ± 4.46	22.65 ± 3.46	−20.22 ± 4.10
4′-O-Methybroussochalcone B/BNA	−13.01 ± 13.17	−4.92 ± 6.90	−17.93 ± 18.65	9.68 ± 11.24	−8.25 ± 8.31
3-Hydroxybakuchiol/BNA	−22.50 ± 3.18	−7.52 ± 7.65	−30.02 ± 8.38	14.48 ± 6.52	−15.54 ± 3.24
Bakuchiol/BNA	−21.74 ± 4.14	−6.69 ± 5.16	−28.43 ± 6.67	12.66 ± 4.32	−15.77 ± 4.44

However, these thermodynamic values should be complemented by the FEL analysis. Although isoneobavaisoflavone (**8**) showed the lowest ΔG_total_ value, its broad and shallow energy landscape (Figure 7A) is suggestive of a more flexible binding pattern than those of the other complexes, despite its favourable ΔG_total_ value. In contrast, bakuchiol (**11**) and 3-hydroxybakuchiol (**10**) exhibited stable and favourable hydrophobic contacts, resulting in predominantly non-polar stabilisation. Their energy balances were consistent with the compact conformations and deep low-energy regions observed in the FEL analysis, indicating robust and stable binding within the catalytic pocket.

Taken together, MM/PBSA and FEL analyses suggest that bakuchiol (**11**) and 3-hydroxybakuchiol (**10**) may represent the more stable complexes among the five ligands when both thermodynamic and conformational features are considered. Although non-polar interactions are a major factor in determining BNA–ligand stability, structural compactness and solvation effects should also be considered to understand the overall binding affinity under dynamic conditions.

### ADMET profiling

The *in vivo* absorption and utilisation of the five selected active compounds have been reported in previous studies, including a pharmacokinetic study from our group^36^^,^^56–58^. Nevertheless, an *in silico* ADMET assessment was performed to provide additional pharmacokinetic and physicochemical context for these BNA target compounds (Table 4). Although the parameters for most of the compounds were generally consistent with Lipinski’s rule-of-five criteria for oral drug-likeness^44^, the lipophilicity of corylifol A (**7**) (log *P* = 5.15) slightly exceeded the recommended threshold (log *P* < 5). This relatively high lipophilicity may be associated with its predicted low aqueous solubility and could contribute to the limited systemic exposure (C_max_ <2.0 ng/mL) reported in previous *in vivo* studies^36^. Bakuchiol (**11**) also showed a single violation (MLOGP = 4.59) but retained favourable predicted GI absorption and membrane permeability. Similarly, isoneobavaisoflavone (**8**), 4′-*O*-methylbroussochalcone B (**9**), and 3-hydroxybakuchiol (**10**) exhibited physicochemical properties broadly consistent with favourable membrane permeability.

**Table 4.** ADMET prediction and drug-likeness assessment of BNA inhibitors from *P. corylifolia.*

ADMET properties	7	8	9	10	11
MW (g/mol)	390.47	322.35	338.40	272.38	256.38
Num. Rotatable bonds	6	1	6	6	6
Num. H-bond acceptors	4	4	4	2	1
Num. H-bond donors	2	1	2	2	1
Molar refractivity	119.25	93.75	100.51	87.44	85.42
TPSA (Å²)	70.67	59.67	66.76	40.46	20.23
Consensus Log *P*_O/W_	5.15	3.64	4.19	4.54	4.92
Solubility: ESOL Class	Poorly soluble	Moderately soluble	Moderately soluble	Moderately soluble	Moderately soluble
GI absorption	High	High	High	High	High
BBB permeant	No	Yes	Yes	Yes	Yes
P-gp substrate	No	Yes	No	No	No
CYP inhibitors	2C19, 3A4	1A2, 2C19, 2C9, 3A4	1A2, 2C19, 2C9, 3A4	1A2, 2C19, 2C9, 3A4	2C19, 2C9
log *K*p (cm/s)	−4.24	−5.57	−4.51	−3.86	−3.52
Lipinski	Yes; 0 violation	Yes; 0 violation	Yes; 0 violation	Yes; 0 violation	Yes; 1 violation: MLOGP > 4.15
Bioavailability Score	0.55	0.55	0.55	0.55	0.55
Bioavailability radar	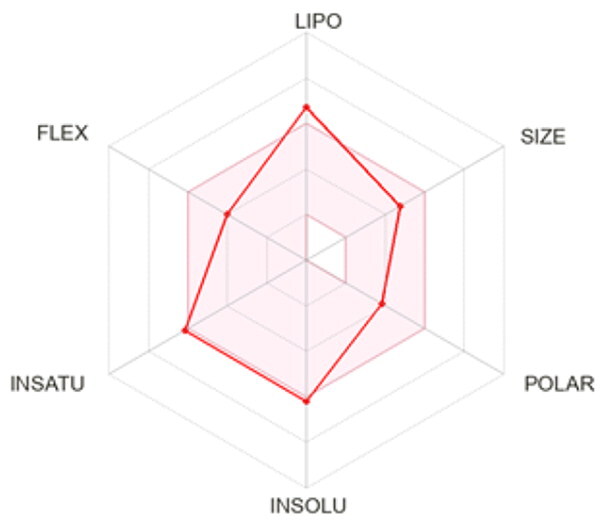	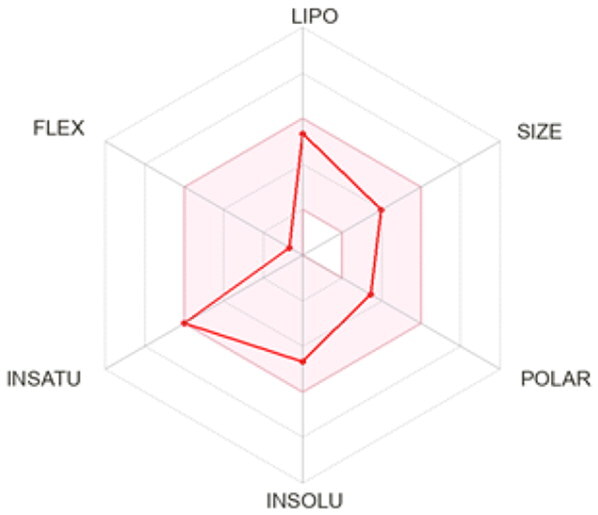	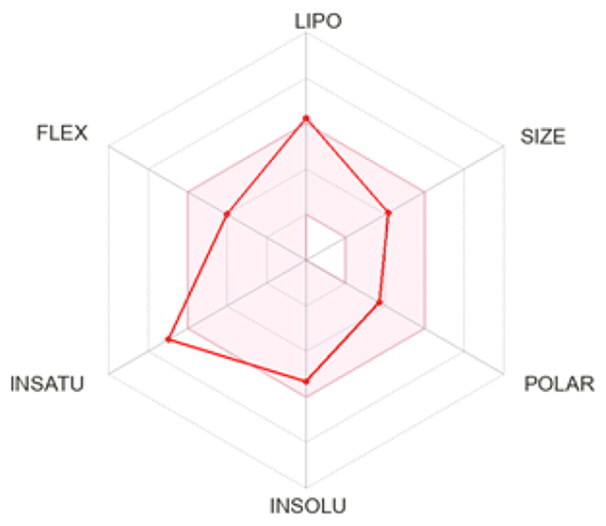	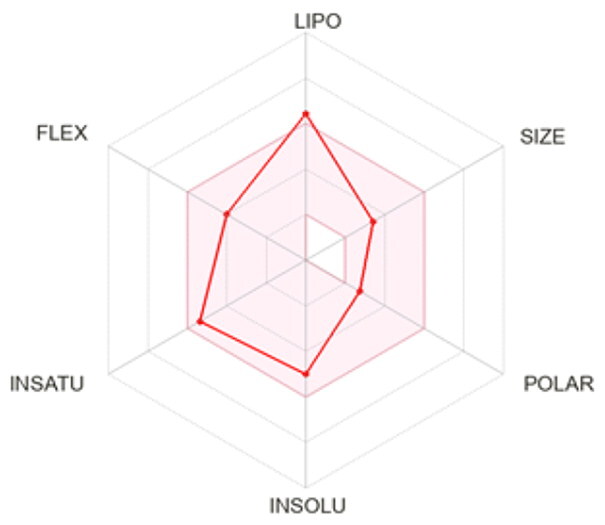	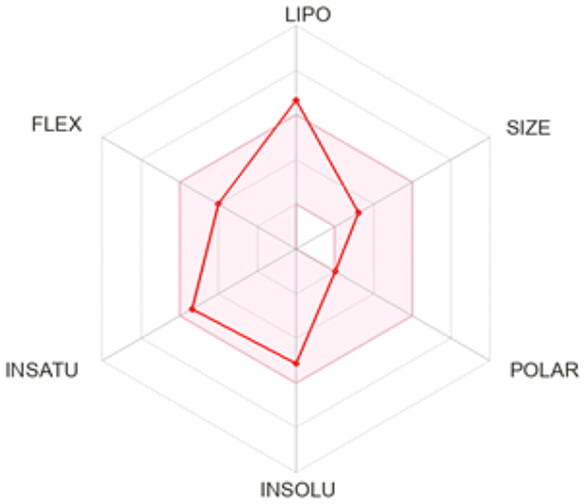
BOILED-Egg (GI & BBB)	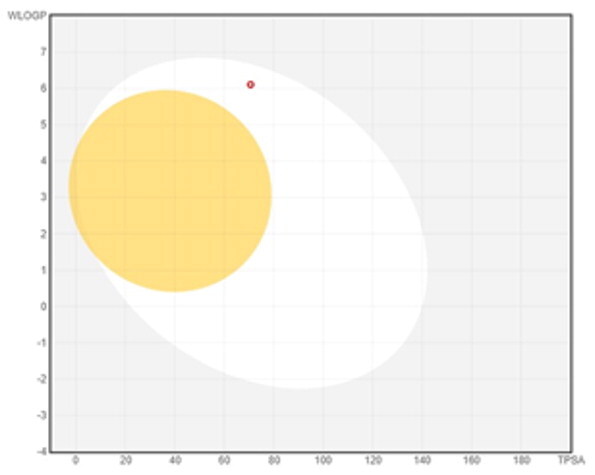	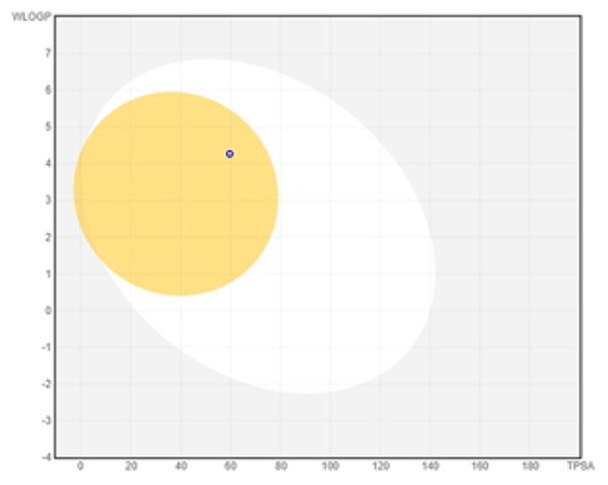	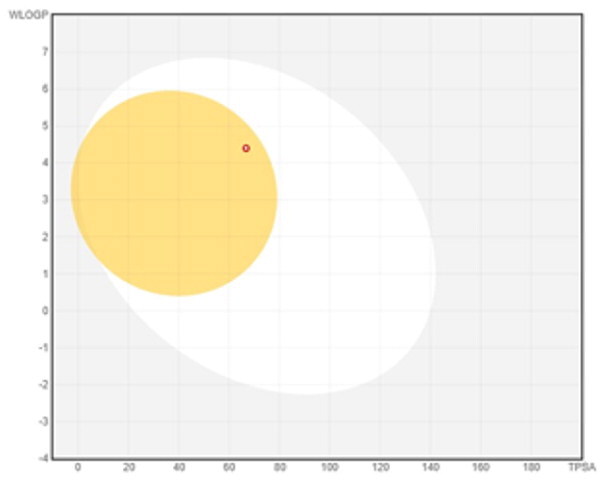	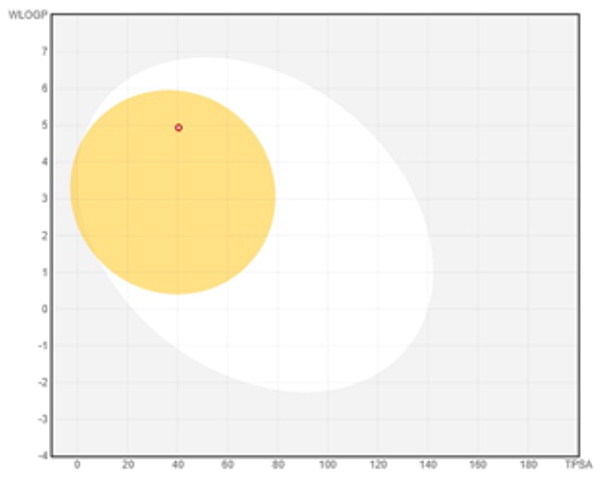	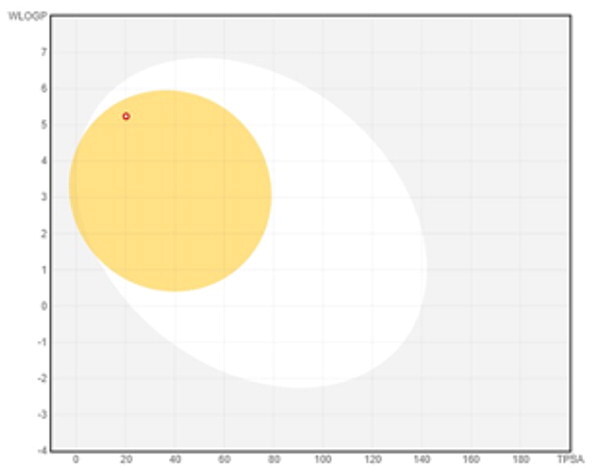

The discrepancy between these favourable *in silico* absorption profiles and the low *in vivo* bioavailability of bakuchiol (**11**) (3.2%)^57^ and 4′-*O*-methylbroussochalcone B (**9**)^36^ highlights the limitations of computational ADMET predictions. Although such models can estimate passive membrane permeability, they may not fully account for factors such as solubility-limited absorption^57^^,^^58^ and metabolic processes, including possible first-pass metabolism^56^^,^^57^. In particular, the low bioavailability of bakuchiol (**11**) appears to be associated with its poor aqueous solubility, which may promote aggregation in aqueous environments^58^, along with relatively rapid systemic clearance (CL = 59.8 ml/min/kg)^57^. Taken together, these factors suggest that solubility and metabolic processes play important roles in determining *in vivo* exposure beyond passive diffusion alone.

All compounds were predicted to exhibit high GI absorption. Four compounds (**8**–**11**) showed potential for BBB permeability with moderate solubility, suggesting possible systemic and central distribution. Moreover, 4′-*O*-methylbroussochalcone B (**9**) exhibited low oral bioavailability but showed relatively prolonged systemic exposure, as evidenced by its sustained plasma levels and a prolonged elimination half-life (14.0–17.0 h) following oral EEPC administration^36^. Several compounds (**8**–**10**) were predicted to inhibit multiple CYP isoforms (CYP1A2, CYP2C19, CYP2C9, and CYP3A4), whereas corylifol A (**7**) and bakuchiol (**11**) showed more limited CYP inhibition profiles.

Overall, the *in silico* ADMET analysis suggests that these compounds exhibit general trends of high predicted gastrointestinal absorption and moderate bioavailability, while also indicating a potential for CYP-mediated interactions. However, these findings should be interpreted with caution, as computational predictions may not fully capture *in vivo* pharmacokinetic behaviour.

## Conclusion

This study is the first to demonstrate the BNA inhibitory potential of *P. corylifolia* using an integrated approach. Through affinity ultrafiltration–UPLC–Q–Orbitrap–MS, 11 representative constituents were prioritised, including putative BNA-interacting candidates that showed time-dependent decreases in peak intensity and stable major compounds included for comparison in the screening workflow. Subsequent enzyme inhibitory assays confirmed that five compounds (**7**–**11**) exhibit potent inhibitory activity (IC_50_ = 4.67–34.09 μM). Notably, corylifol A (**7**), 4′-*O*-methylbroussochalcone B (**9**), 3-hydroxybakuchiol (**10**), and bakuchiol (**11**) showed superior potency (IC_50_ = 4.67–11.27 μM) than the positive control, quercetin (IC_50_ = 13.63 ± 1.05 μM). Enzyme kinetic analysis identified bakuchiol (**11**) as a competitive inhibitor, whereas compounds **7**–**10** acted as mixed-type inhibitors. Molecular docking, FEL, and MM/PBSA analyses further suggested that bakuchiol (**11**) and 3-hydroxybakuchiol (**10**) may form relatively stable BNA complexes when both thermodynamic and conformational features are considered. Although isoneobavaisoflavone (**8**) showed the most favourable MM/PBSA binding free energy, its broad and shallow FEL profile indicated greater conformational flexibility than the other ligands. Taken together with its inhibitory activity and predicted ADMET profile, bakuchiol (**11**) appears to be a promising lead candidate among the tested compounds. Overall, these findings provide useful insights into the BNA inhibitory potential of *P. corylifolia* constituents and support the value of an integrated approach for identifying bioactive components from complex natural extracts.

## Supplementary Material

Supplementary Materials.docx

## Data Availability

The data that support the findings of this study are available from the corresponding author upon reasonable request.
