# Supplementary material for: Systematic identification of bacterial neuraminidase inhibitors from Psoralea corylifolia using ultrafiltration-UPLC-Q-Orbitrap-MS and molecular dynamics
Source: J Enzyme Inhib Med Chem. 2026 May 25;41(1):2676094. doi: 10.1080/14756366.2026.2676094 (PMC13202655; doi:10.1080/14756366.2026.2676094)
Supplement: Supplementary Materials.docx [file IENZ_A_2676094_SM8791.docx]

**Supplementary Materials**

**Systematic identification of bacterial neuraminidase inhibitors from *Psoralea corylifolia* using ultrafiltration-UPLC-Q-Orbitrap-MS and molecular dynamics**

**
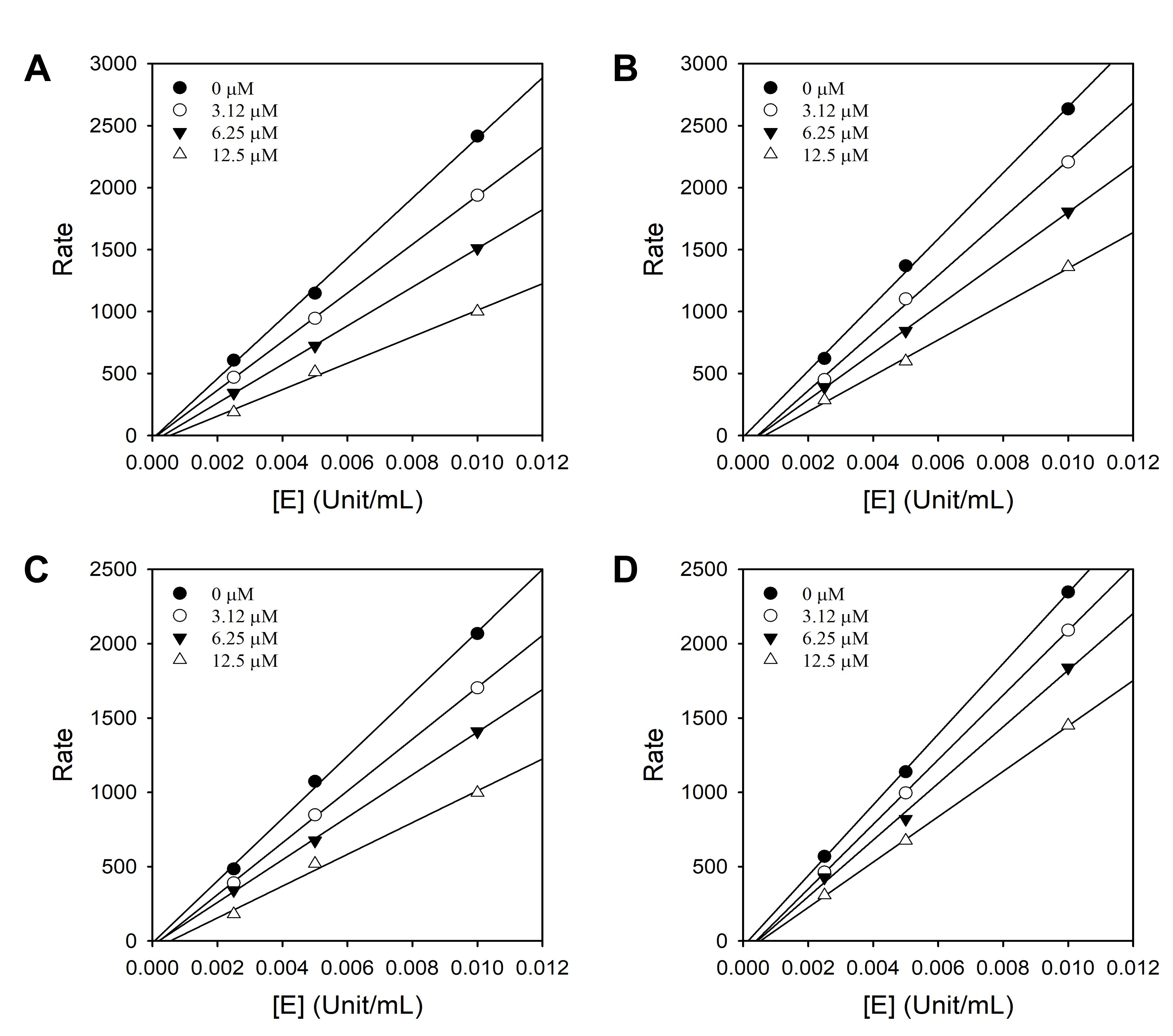
**

**Figure S1.** Enzyme concentration-dependence of BNA catalytic activity at various concentrations of compound **7** (A), **8** (B), **10** (C), and **11** (D).

**
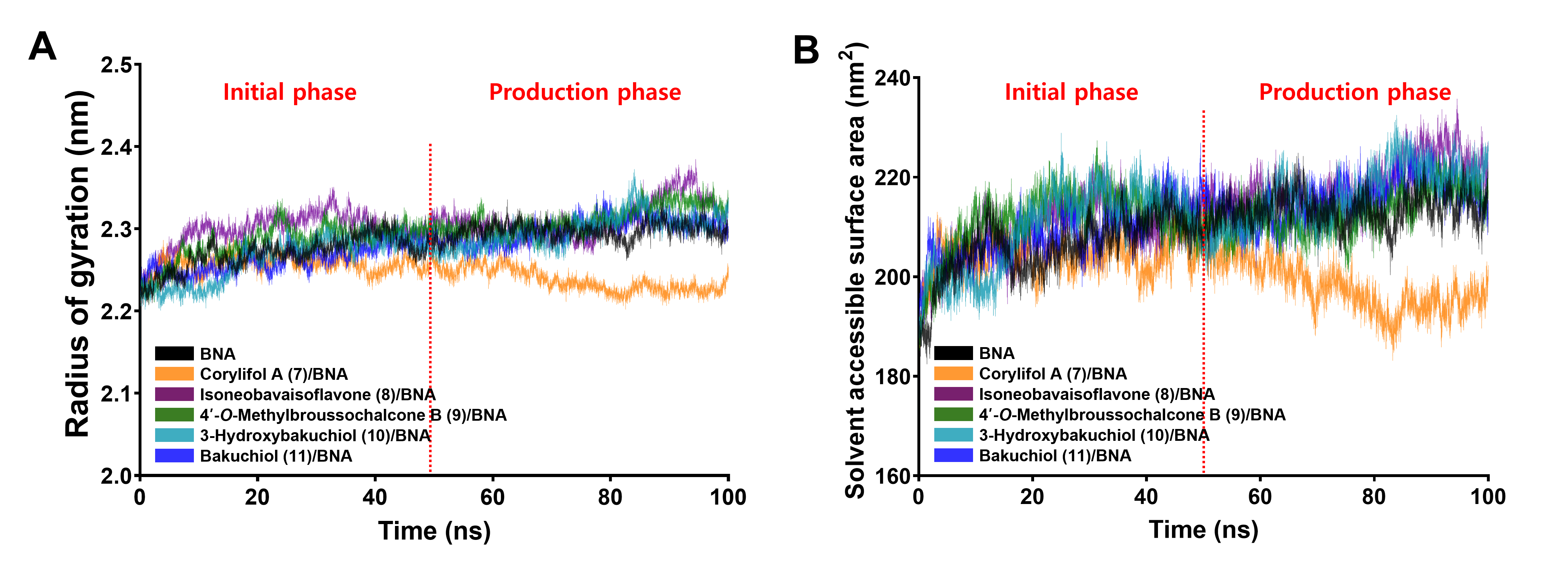
**

**Figure S2.** Radius of gyration (Rg) and solvent-accessible surface area (SASA) of BNA in free and ligand-bound states during 100 ns MD simulations. (A) Rg profiles of free BNA and BNA–ligand (**7**–**11**) complexes. (B) SASA profiles of BNA for the same systems.

**
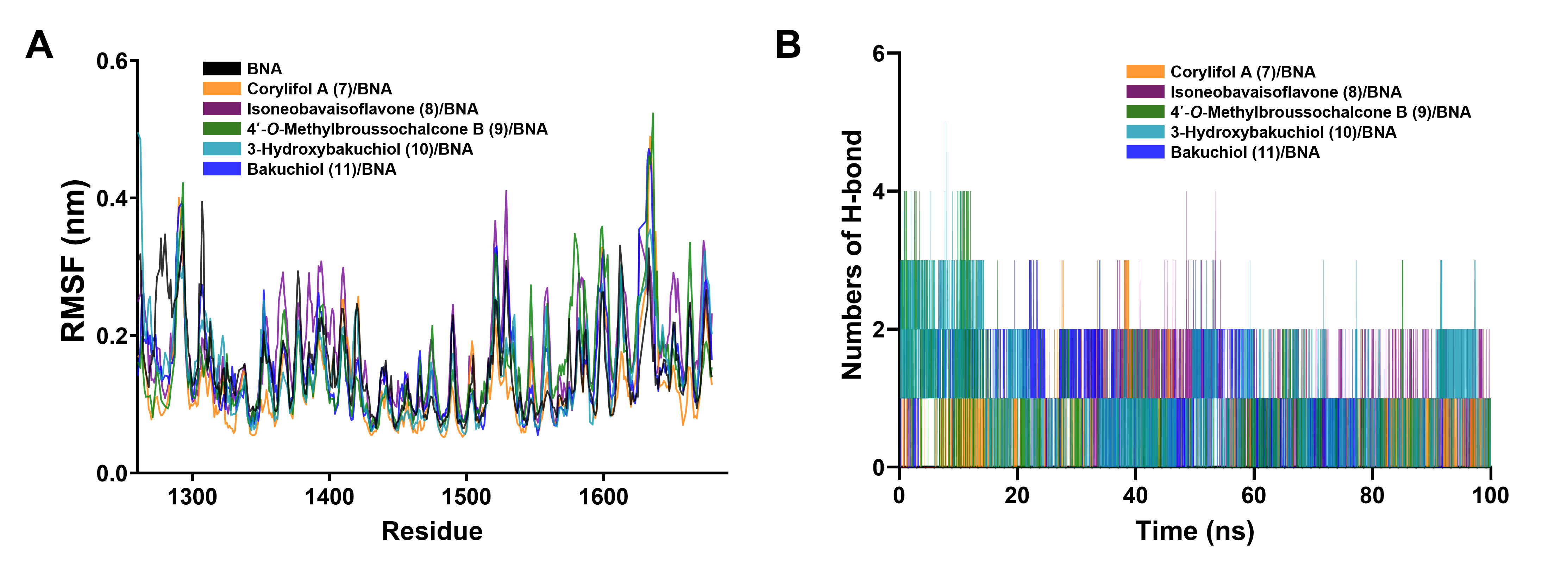
**

**Figure S3.** Residue flexibility and hydrogen-bond dynamics of BNA. (A) RMSF profiles of free BNA and complexes with compounds **7**–**11**. (B) The number of hydrogen bonds between BNA and each ligand over 100 ns, indicating the persistence of polar interactions.

**
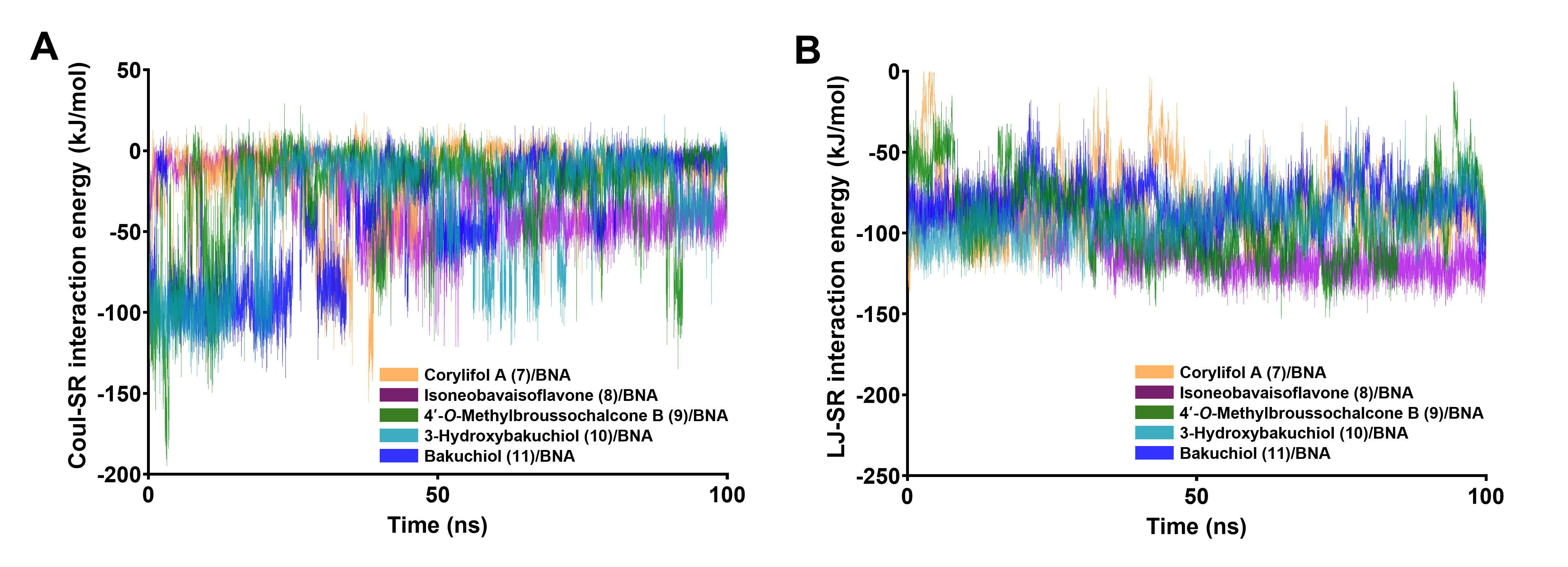
**

**Figure S4.** Non-bonded interaction energies between BNA and ligands **7**–**11**. (A) Coulombic short-range (Coul-SR) electrostatic interaction energies for BNA–ligand complexes. (B) Lennard–Jones short-range (LJ-SR) van der Waals interaction energies for BNA–ligand complexes.
